# Precision Delivery Using Nanopipette for Single-cell Studies

**DOI:** 10.61558/2993-074X.3488

**Published:** 2024-08-21

**Authors:** He Zhang, Md Maksudur Rahman, Yang Tao, Joseph W. Sampson, Hang Ren

**Affiliations:** a Department of Chemistry, The University of Texas at Austin, Austin, TX 78712, USA; b Center for Electrochemistry, The University of Texas at Austin, Austin, TX 78712, USA; c Texas Materials Institute, The University of Texas at Austin, Austin, TX 78712, USA

**Keywords:** Scanning ion conductance microscopy, Nanopipette, Single cell image, Local delivery, 扫描离子电导显微镜, 纳米移液管, 单细胞成像, 局部递送

## Abstract

Nanopipette based scanning probe technique is a versatile tool in non-contact imaging in biology. In addition to the topographic imaging, its capability of localized delivery of bio-active molecules is emerging. In this mini review, we introduce the applications of nanopipette in single-cell researches with a focus on localized delivery. The working principles of three delivery modes including resistive pulse, pressure-driven flow, and electroosmotic flow-driven delivery are summarized and compared. Their applications in single-cell researches are reviewed. The current technical challenges in scanning ion conductance microscopy-based delivery, and their growing influence in medicine and pharmacologic researches are also discussed.

## Graphical Abstract

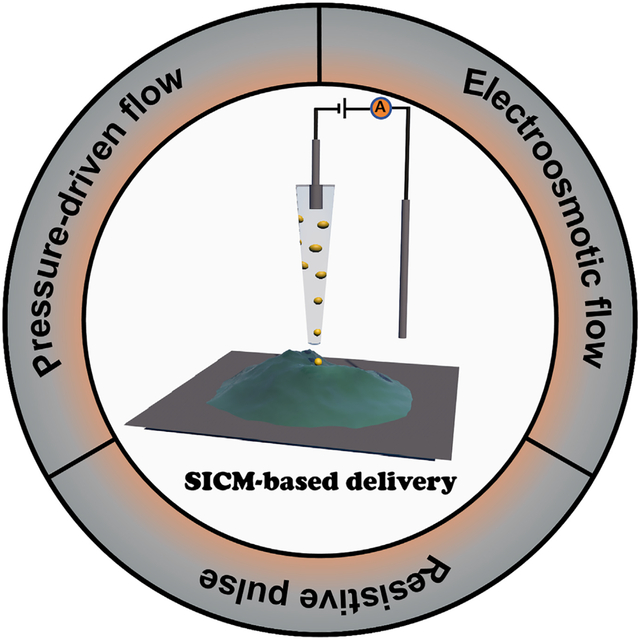

## 摘要：

基于纳米移液管的电化学扫描探针技术是一种多功能非接触成像工具, 并被广泛应用于生物学研究。 除了一般的表面形貌成像研究外, 它在局部递送生物活性分子方面的潜力也逐渐显现。 在这篇简短综述中, 我们介绍了这种技术在单细胞研究中的应用, 特别是局部递送。 我们总结并比较了三种递送模式的工作原理, 包括阻抗脉冲、 压力驱动和电渗流驱动递送, 还回顾了这些模式在单细胞研究中的应用。 此外, 本文还讨论了基于扫描离子电导显微镜的递送技术所面临的技术挑战及其在医学和药理学研究中日益增长的影响力。

## 1. Introduction

Cellular heterogeneity on the genomic, transcriptomic, proteomic, and metabolomic levels is closely associated with understanding how individual cells process information and responding to perturbations, particularly in comprehending differences under physiological and pathophysiological conditions [1–3]. Traditional analysis of biological samples often fails to capture the full complexity of cellular behavior. These methods often assume that cells of the same type behave identically, which is not always the case. Moreover, they have inherent limitations like low resolution, contrast issues, and difficult observing dynamics and quantitative analysis, especially in heterogeneous samples. This is particularly relevant in scenarios involving molecular transport, such as drug delivery within cellular membranes or ionic flow through dendrites of neuronal cells.

Deciphering the relationships between human diseases and electron transfer, energy conversion, and substance transformation in biological processes has been recently proposed among the top ten scientific challenges in electrochemistry [4]. Understanding this question will inevitably involve the disintegration of heterogeneity among a population of cells. Electrochemical techniques are uniquely positioned to address some of these questions because the electron transfer and energy conversion in cells are electrochemical. Scanning electrochemical techniques, such as Scanning Electrochemical Microscopy (SECM) and Scanning Ion Conductance Microscopy (SICM), have been developed to offer further spatial and temporal resolution in electrochemical measurements [5–10]. These techniques utilize a probe that not only can measure the topography of the cells, but also can probe the local chemical information without making contact with the sample. In SECM, the local concentration of redox-active molecules, e.g., ${\text{O}}_{2}$ or other metabolites, can be measured electrochemically using a microelectrode or nanoelectrode as the probe [11–15]. This nanoprobe is scanned across the surface of the sample to map the chemical information, offering invaluable insights into the dynamic electrochemical processes at play. For example, Wu et al. developed a fluorinated monolayer-modified Pt ultra-microelectrode for detecting NO released by a single MCF-7 cell under cadmium stimulation with high sensitivity on the cell surface [16]. Nebel et al. adopted a Pt nanoprobe to image the respiration activity at a HEK293 cell by monitoring the currents of oxygen reduction reaction (ORR) [17]. We will not review SECM here and the readers are referred to some comprehensive reviews [5].

**Scheme 1.**
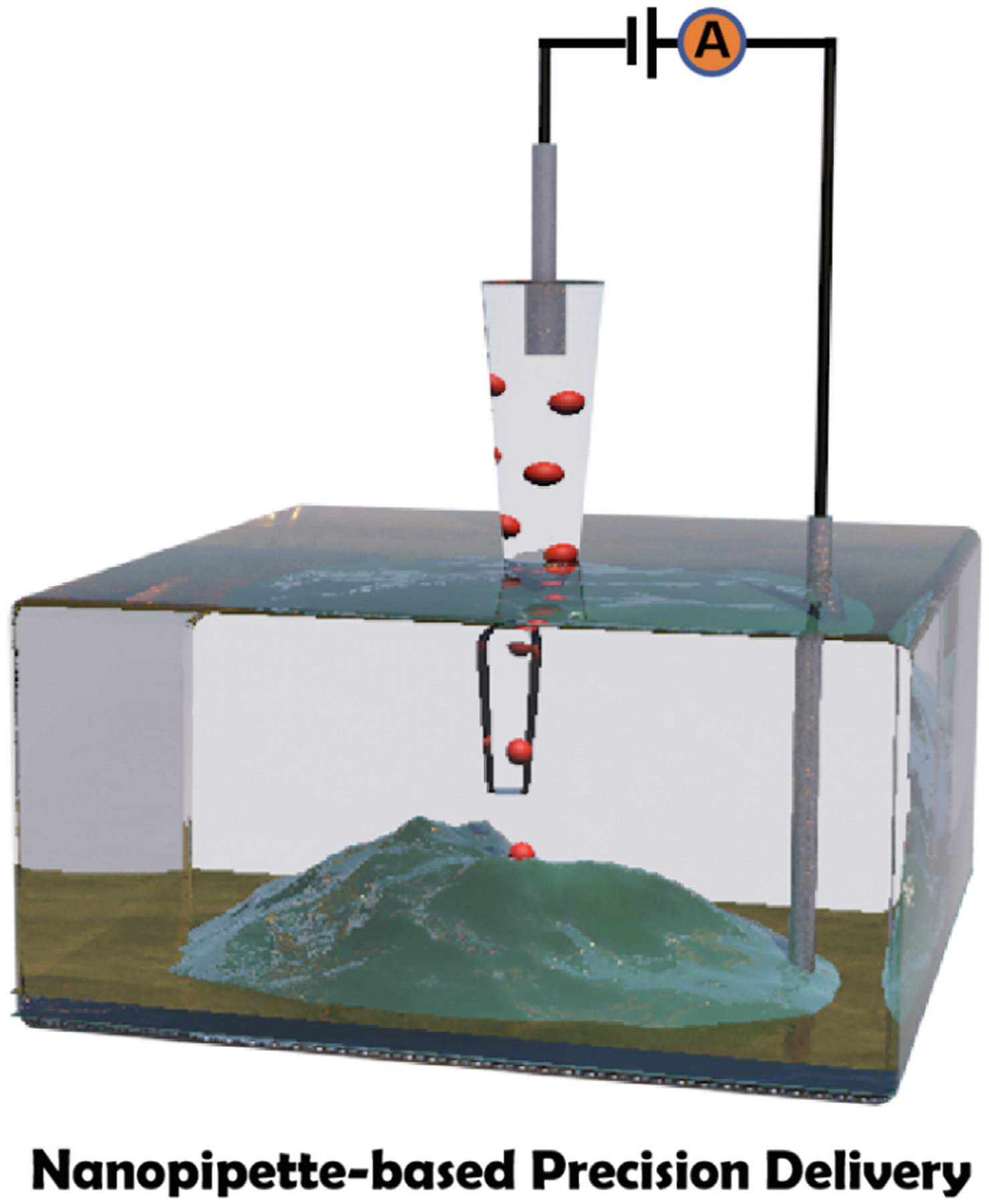
Nanopipette-based precision delivery on single-cell researches with three delivery modes of resistive pulse, pressure-driven flow, and electroosmotic flow-driven delivery.

Scanning Ion Conductance Microscopy (SICM) is another scanning probe technique primarily employed for mapping the topography of various substrates [18,19]. The probe is a glass capillary with a nanometer-size opening, often called nanopipette. Because this review will focus on the nanopipette, we will briefly describe the principle of this technique. For detailed principles and examples, the readers are referred to a recently published comprehensive review [8]. The nanopipette is filled with electrolyte and immersed in the bath electrolyte solution (e.g., cellular medium), where the samples, e.g., cell cultures, are placed. A potential is applied between a quasi-reference counter electrode (QRCE) placed inside the nanopipette and another QRCE placed in bath solution, and the ionic current is recorded (Scheme 1). This ionic current is mainly determined by the access of ions through the nanoscale opening, and is sensitively dependent on the presence of a surface near the tip. This current feedback allows detailed topographic imaging of the sample submerged in solution, which is especially suitable for biological samples because the imaging can be made without physical contact [20,21]. For example, the SICM technique enables the exploration of single-cell behavior such as membrane dynamics [22], cell motility [23], tight junctions [24], and secretory events [25] under conditions that mirror natural cellular conditions. Combining SICM with super-resolution optical fluctuation imaging (SOFI) allows visualizing actin dynamics in living cells with subdiffraction-limited resolution [26].

In addition to chemical mapping, the nanopipettes are also well suited to chemically perturb the biological samples locally by controlled deliveries of chemicals and biomolecules, which is the topic of this mini review. In the following, we will introduce the fundamental principles underlying the delivery using pressure-driven flow and electroosmotic flow, as well as resistive pulse sensing (Scheme 1). We also highlight the significant research advancements in the localized delivery for single-cell studies implanting these techniques. Lastly, we share our perspective on the challenges and innovations of these techniques and their possible applications in medicine and pharmacology.

## 2. Pressure-driven flow-based delivery

**Fig. 1.**
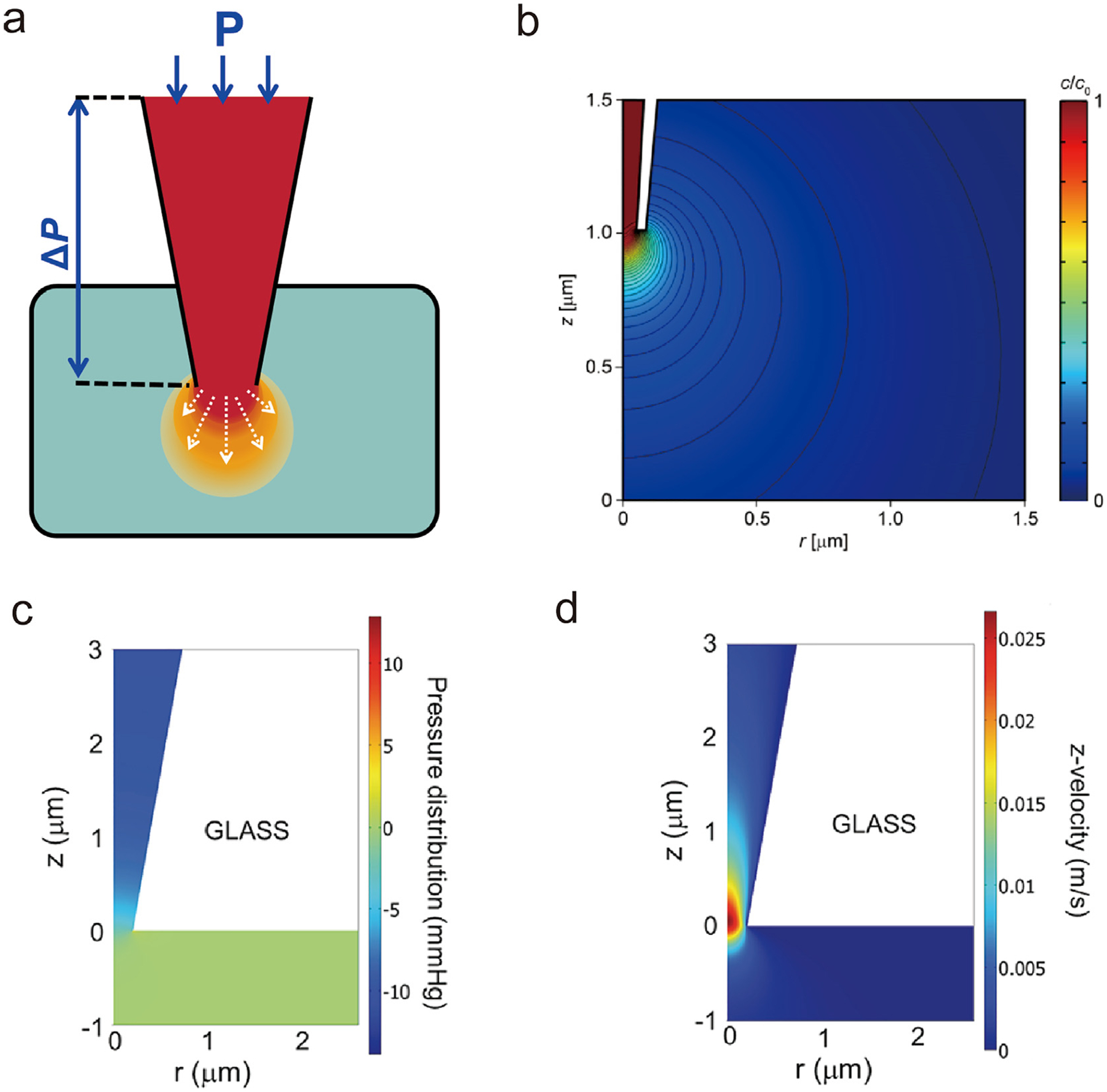
(a) Schematic diagram of pressure-driven flow-based delivery. (b) Magnitude and contour profiles of the concentration when a pressure of 20 kPa is applied at the top of the nanopipette [29]. (c–d) Simulated pressure and fluid velocity in the z direction through a nanopipette orifice with an applied pressure of 10 mmHg in the nanopipette [30].

Pressure-driven flow in nanopipette involves applying a hydrostatic pressure differential (Δp) to induce fluidic through the opening of the nanopipette. This flow can facilitate controlled mass transport of the chemicals, as illustrated in Fig. 1a. Positioning the probe in the SICM mode offers spatial resolution for delivery at specific location of interest. The mass transport of delivered molecules can be described by the Nernst–Planck equation (1):

(1) $$J=-D\nabla C+Cu_{p}$$

Where D is the diffusion coefficient, C is the concentration of the species of interest and $u_{p}$ is the velocity of the solution under pressure-driven flow. The fluid flow rate $Q_{\text{tot},\Delta p}$ due to an applied hydrostatic pressure Δp, can be obtained as [27]:

(2) $$Q_{tot,\Delta p}=\frac{3\pi R_{0}^{3}\text{tan}(\theta )\Delta p}{8\eta }$$

In Eq. 2, $R_{0}$ and θ are the opening radius and half-cone angle of the nanopipette, respectively, η is the solution viscosity. The fluid velocity centered at the orifice of the nanopipette ($v_{p}$) can be calculated as [28]:

(3) $$v_{p}(R)=\frac{Q_{\text{tot},\Delta p}}{4\pi R^{2}}$$

The delivery time t for an analyte particle to travel from point α to point β can be calculated by integrating the inverse of equation 4:

(4) $$t=\int_{\alpha }^{\beta }v^{-1}dR=\frac{4}{3}\pi Q_{tot,\Delta p}\left(\alpha^{3}-\beta^{3}\right)$$

Similarly, the concentration profile of the molecules near the pipette opening, as well as the amount of species delivered through the pipette in a given time can be obtained by solving the Nernst-Planck equation with appropriate boundary conditions [29].

Based on the pressure-driven flow in a nanopipette, Korchev and coworkers demonstrated the delivery of a specific analyte through a nanopipette for SICM experiments [29]. Their work validates the prediction of the analyte concentration profile, which depends on both the applied voltage/pressure and the distance from the surface. As shown in Fig. 1b, finite element simulation results illustrate a spherical concentration gradient of the analyte as it is emitted from the nanopipette. White and coworkers also performed finite element simulations of pressure-driven flow through a nanopore [30]. The pressure remains constant and equivalent to the applied pressure at distances >3μm from the orifice into the nanopipette. In comparison, the pressure distribution across the bulk solution remains constant and equivalent to the exterior pressure at distances <-0.5μm (0μm is the orifice) (Fig. 1c). However, the fluidic pressure decays rapidly from the bulk solution through the orifice, indicating that the geometry of the orifice influences the fluid flow. At positions relatively far from the orifice, the fluid velocity is 0, indicating that the fluid is still while the velocity increases only across both sides. It should be noted that the fluid velocity changes more rapidly upon exiting the bulk through the orifice than from the nanopipette through the orifice (Fig. 1d). Baker et al. took an opposite approach by controlling the driving pressure of the nanopipette to collect cytoplasm from single *Allium cepa* cells, and combined with matrix-assisted laser desorption ionization mass spectrometry (MALDI-MS) for a detailed analysis of its components [31]. Besides, they also found that long-shank pipettes exhibited a linear relationship between sampled volume and pressure, making them more advantageous for small volume sampling experiments.

## 3. Electroosmotic flow-driven delivery

**Fig. 2.**
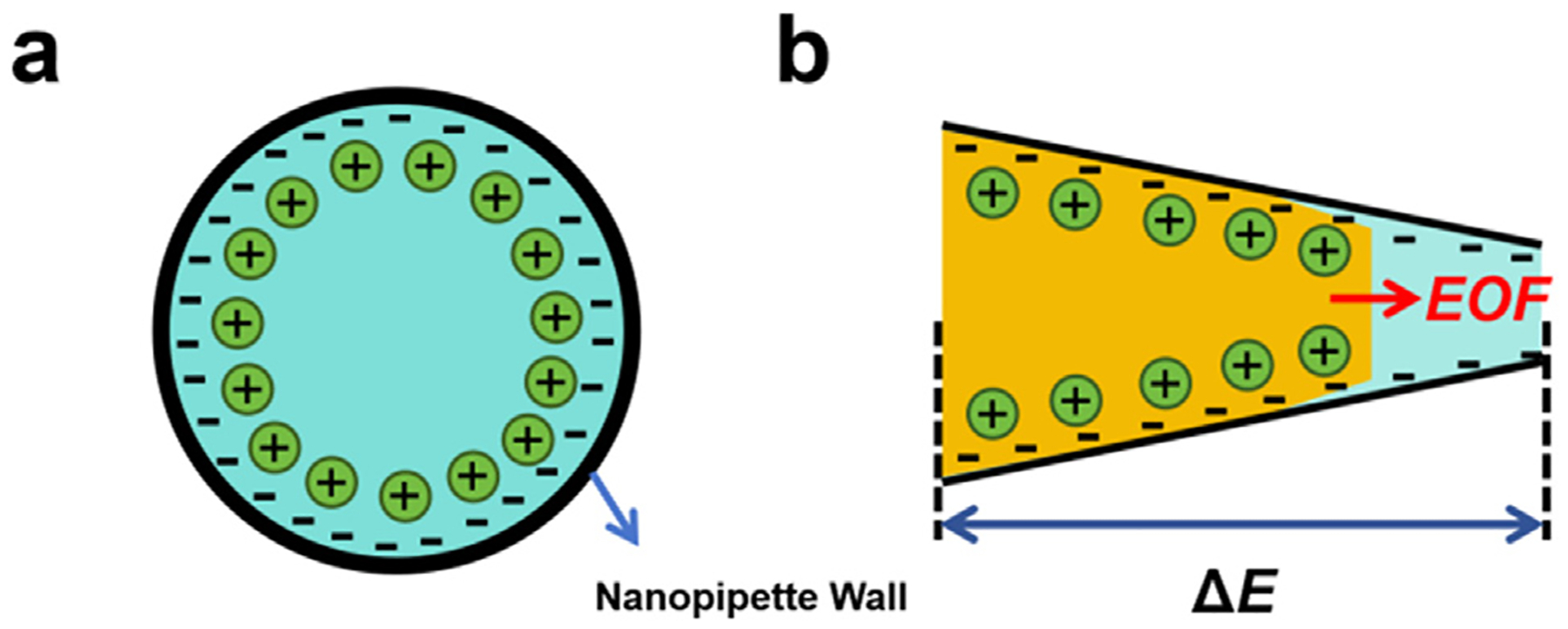
(a) Electrical double layer of nanopipette wall. (b) Electroosmotic induced flow.

Electroosmotic flow (EOF) is another type of flow that can be generated readily in a nanopipette. EOF is induced by the interaction between the externally applied electric field and the excess charges within the electrical double layer (EDL). The use of EOF is very effective in controlling the motion of analytes in micro- and nano-channels. The surface of glass nanopipettes contains abundant silanol groups (Si–OH), which can be deprotonated to form fixed negative charges. An excess cation will be attracted near the interface in the solution, creating the EDL (Fig. 2a). The electric field through the orifice caused by the applied potential across the pipette forces the excess mobile charge in the EDL to move, which drags the solvent to create EOF (Fig. 2b). The velocity field can be described by solving the Navier–Stokes equation coupled with the Nernst Plank–Poisson equation. The magnitude of the EOF velocity ($v_{\text{EOF}}$) can be simplified without explicit treatment of the EDL as the Helmholtz–Smoluchowski equation [32]:

(7) $$v_{\text{EOF}}=-\frac{\varepsilon \varepsilon_{0}\zeta }{\eta }E_{x}$$

where ε is the relative dielectric constant of the solution, $\varepsilon_{0}$ is the vacuum permittivity, η is the viscosity of the solution, and $E_{x}$ is the strength of the applied electrical field tangent to the surface. Zeta potential, is the potential at a distance from the capillary wall in the EDL called the shear plane [33]. Zeta potential is directly affected by the surface charge of the capillary wall and the compactness of the EDL. Unlike a pressure-driven flow, EOF exhibits a distinctive, uniform “plug flow” velocity profile outside the double layer, making it highly efficient for the controlled fluid movement in micro- and nano-scale applications.

**Fig. 3.**
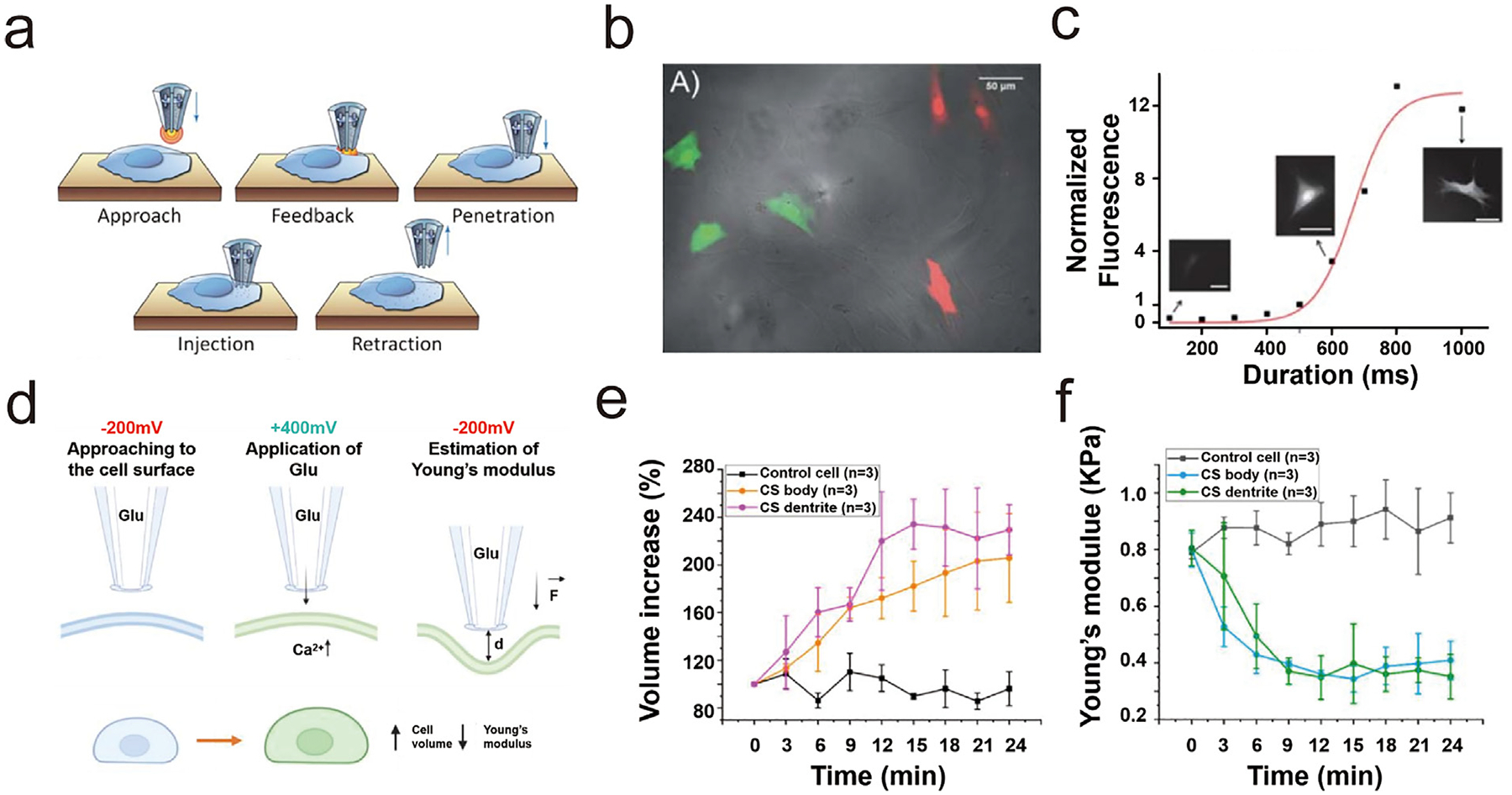
(a) Schematic illustration of cell surface detection, penetration, and injection using a double-barrel nanopipette [35]. (b) Stacked images of human fibroblast cells injected with a double-barrel nanopipette containing CFSE (green) and SR (red) in separate barrels [35]. (c) Fluorescent quantification of the injected carboxyfluorescein in human BJ fibroblasts [35]. (d) Local delivery of glutamate to a neuronal cell and measurement of cell mechanics. Left: approach at −200 mV. Middle: glutamate release at +400 mV. Right: Estimation of Young’s modulus [36]. (e) Cell volume increase and (f) mean value of Young’s modulus after local delivery of glutamate [36].

Bruckbauer et al. demonstrated the delivery of individual fluorescently labeled probe molecules to the plasma membrane using a nanopipette with EOF [34]. Combining the delivery with single-molecule fluorescence tracking shows several advantages, including the targeted application of probes to specific membrane regions, controlled release of a small number of molecules, low background noise in combination with total internal reflection fluorescence microscopy, and the ability to optimize and repeat experiments on the same cell. Serger and co-workers also quantified the delivery of fluorescent molecules into single cells using a double-barrel nanopipette [35]. Fluorescence images show that the fluorescence intensity of carboxyfluorescein succinimidyl ester (CFSE) in the human BJ fibroblasts increased in a sigmoidal fashion with increasing duration of the applied voltage (Fig. 3a–c). Bruckbauer et al. used similar methods to deliver fluorophore-labeled probe molecules to defined regions of the cell surface. They measured localized diffusion coefficients via simultaneous single-molecule fluorescence tracking [34]. Different diffusion coefficients were revealed, e.g., $0.79\pm 0.04\;\mu {\text{m}}^{2}\cdot {\text{s}}^{-1}$ in the acrosomal region and $0.10\pm 0.02\;\mu {\text{m}}^{2}\cdot {\text{s}}^{-1}$ in the postacrosomal region, suggesting different membrane structures in these two regions.

The EOF-based local delivery using a nanopipette can also reveal the response of single cells to local chemical stimuli. For example, Kolmogorov et al. uncovered that glutamate stimulation in hippocampal neurons leads to notable changes: an increase in cell volume and a reduction in Young’s modulus (Fig. 3d) [36]. Leveraging the precise localization capabilities of SICM in their morphological analysis of these neurons, they found that the dendrite of the cells responded more significantly to glutamate stimulation (Fig. 3e and f). Page et al. developed a dual-channel scanning probe nanopipette that enabled simultaneous SICM and SECM measurements for a *Zea mays* root hair cell [37]. Here, SICM was used to deliver ${\text{Ru}{\left({\text{NH}}_{3}\right)}_{6}}^{3+}$ locally while the SECM was used to detect the local concentration of ${\text{Ru}{\left({\text{NH}}_{3}\right)}_{6}}^{3+}$, demonstrating faster uptake of ${\text{Ru}{\left({\text{NH}}_{3}\right)}_{6}}^{3+}$ at the root hair tip compared to the cell body. This setup is particularly useful to study the different rates of molecular uptake at different regions with nano-scale resolution.

## 4. Resistive pulse-driven delivery

**Fig. 4.**
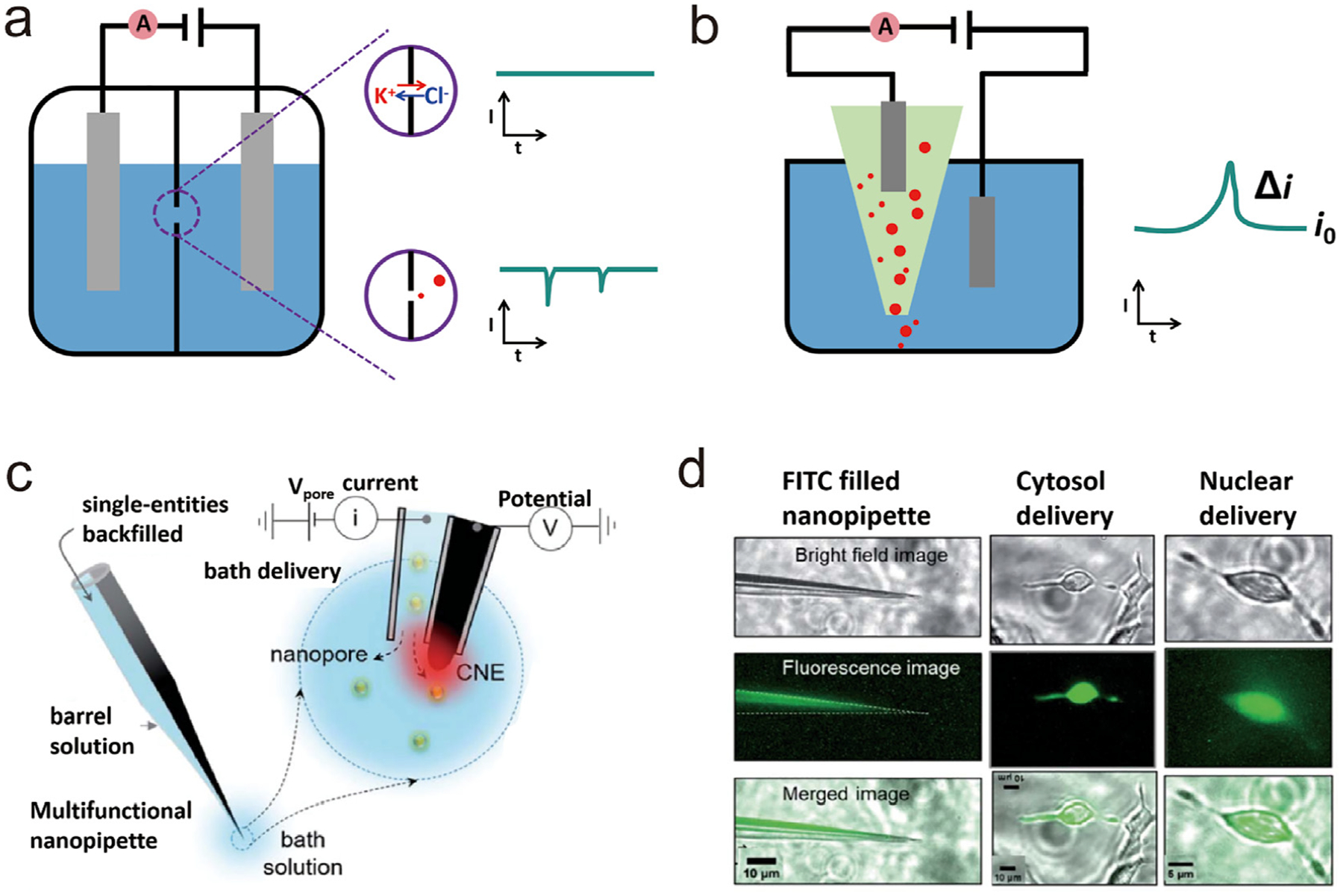
(a, b) Schematic illustrations of the working principles of (a) Coulter-counting and (b) SICM-based resistive pulse-driven delivery. (c) Schematic of the experimental setup of the bath delivery and single-entity detection via a multifunctional long tapered nanopipette [40]. (d) Optical images of cytosol and nucleus deliveries in HEK293 cells [40].

Resistive pulse is a method that allows the measurement of the size of the particle, which originates from the Coulter counter but can be utilized for controlled delivery of particles. The principle is based on the blockage of ionic current in a channel or nanopore by each translocating particle, resulting in a decrease in the current transient [38]. As depicted in Fig. 4a, a standard Coulter counter comprises two electrolyte-filled chambers linked by a narrow channel. A potential is applied between the two compartments, and the resulting current is limited by the ionic transport through the aperture. When analytes with a size smaller but comparable with the dimension of the aperture are introduced into one chamber, their transit through the aperture can momentarily block the pore, resulting in short spikes of altered resistance. The frequency of the spike is related to the concentration of particles, while their amplitude correlates with their size. The nanopipette can effectively serve as a resistive pulse sensor. For the application in delivery, instead of sensing the particles in the solution, the setup can be used to count the number of particles delivered out of the nanopipette. Essentially, when a potential difference is applied between two combined reference/counter electrodes (one located inside the nanoprobe and the other one outside), analytes are driven from the nanopipette to the external solution. A current spike should occur every time when a particle is ejected from the nanopipette, enabling precise control of the number of analytes delivered (Fig. 4b) [39].

Pandey et al. engineered a versatile nanopipette equipped with both a nanopore and a nanoelectrode (made of pyrolytic carbon) at its apex (Fig. 4c), which achieved controlled intracellular delivery at the single-cell level with precise single-entity counting [40]. They successfully administered gold nanoparticles, FITC dye molecules, and ferritin proteins into HEK293 cells and cardiomyocytes. The efficacy of these deliveries was verified by the increased scattering and fluorescence signals, as illustrated in Fig. 4d. Very recently, Actis et al. demonstrated the nanopipette could be used for both cell lines and primary cells to perform quantitative deliveries of DNA, globular proteins, and protein fibrils, all at single molecule resolution, and that the macromolecules retained function after delivery into the cells [41]. Besides, combining experiments and computational modeling, they also proved that macromolecular crowding in the cell increases the signal-to-noise ratio for the detection of translocation events.

## 5. Summary and outlook

In this mini review, the working principles of pressure-driven flow, resistive pulse, and electroosmotic flow-driven delivery methods are introduced, along with a summary of their recent developments in single-cell researches. Pressure-driven flow is the most versatile and easy to operate, and suitable for various substances by controlling the pressure difference to push the solution through the pore of nanopipette. However, it is generally efficient in micro-to nano-scale channels. In contrast, electroosmotic flow (EOF)-driven delivery has developed more rapidly because it excels in flow control in the nanoscale, and its control can be simply through the control of applied potential. Consequently, it is widely favored in the research of delivering charged ions, molecules, and/or nanoparticles. The resistive pulse method, while not a flow method, offers the highest sensitivity from significant signal amplification, allowing counting particles with single digit precision. Therefore, resistive pulse is ideal for single-molecule or single-particle deliver when the delivery target has a comparable size with the nanochannel [42–44]. Reproducibly designing a solid state nanopipette with molecular dimension and structure remains a challenge for the further development of resistive pulse-based delivery using nanopipettes.

Based on these delivery modes, nanopipettes have enabled SICM imaging to study the dynamics of living cells, and recently emerged as a promising tool for precise localization and chemical delivery, as well as extraction of cellular components. These exciting capabilities have provided valuable insights into cellular behaviors and dynamics that are often altered or lost in non-physiological conditions. For example, a recent study demonstrated that nanopipettes allow the selective isolation of small subsets of mitochondria from individual living cells to quantify the mutant mitochondrial genomes by high-throughput DNA/RNA sequencing [45]. Such insights are crucial for unraveling the mechanisms behind the gradual accumulation of cellular degenerative mutations, which are known to lead to cellular dysfunction and, eventually, cell death. In the future, combining these electrochemical strategies with spectroscopy, microscopic imaging, force, and magnetic measurement techniques will contribute to multi-scale, multi-mode, and highly sensitive new characterizations of biological systems [1].

Here, we provide a few perspectives for the further development of nanopipette-based techniques for cellular studies.

### (1) Temporal resolution

The nanopipette-based single-cell study can be benefitted by further improving the temporal resolution of SICM imaging. Some designs and principles from the field of high-speed atomic force microscopy (HS-AFM) can be borrowed to further improve the temporal resolution in the non-contact scanning mode, which could allow the study of faster cellular dynamics [46].

### (2) Precision control by understanding the transport in nanochannels.

The spatial resolution of delivery can be further improved by using a smaller nanopipette. While EOF and pressure-driven flow have demonstrated the delivery of molecules intra and extra-cellularly, the exact amount of delivery often needs calibration, especially when the channel becomes narrow. A more detailed model that allows precise prediction of the role of electrophoresis (i.e., migration) and electroosmotic flow, especially when the width of the nanopipette approaches the thickness of the EDL, would be important when delivery is performed at high spatial resolution and low ionic strength. On the other hand, new mechanisms that can further focus on the molecules and ions can be explored to further improve the precision and spatial resolution.

### (3) Higher throughput

The throughput of delivery using a single nanopipette is currently low. Development of multichannel devices, or improvement of the speed of nanopipette manipulation could potentially increase the throughput of the experiment. It is also desirable to develop automated systems that can meticulously manage probe positioning, precision-targeted molecule delivery, and comprehensive data acquisition with minimal human intervention. Besides, the integration of SICM with other advanced high-throughput technologies, such as microfluidics or array-based platforms, represents a promising avenue.

### (4) Applications in medicine and pharmacology

Nanopipette offers a highly precise method for drug delivery, targeting specific cells or tissues with remarkable accuracy, which can be used for screening of therapeutic material. In addition, targeted delivery of potential pharmaceutical compounds directly to distinct cells or specific intracellular compartments enables a precise evaluation of drug efficacy and toxicity, allowing enhanced understanding of drug-cell interaction at the cellular level.
